# Distribution of Primary Resistance Mutations in Saint Petersburg in Patients with Chronic Hepatitis C

**DOI:** 10.3390/diagnostics12051054

**Published:** 2022-04-22

**Authors:** Diana Valutite, Yulia Ostankova, Alexandr Semenov, Liudmila Lyalina, Areg Totolian

**Affiliations:** 1 Saint Petersburg Pasteur Institute, 197101 St. Petersburg, Russia; shenna1@yandex.ru (Y.O.); lyalina@pasteurorg.ru (L.L.); pasteur@pasteurorg.ru (A.T.); 2 FSBI State Scientific Center of Virology and Biotechnology «Vector» of the Federal Service for Surveillance of Consumer Rights Protection and Human Welfare (Rospotrebnadzor), 620030 Ekaterinburg, Russia; alexvsemenov@gmail.com

**Keywords:** hepatitis C virus, resistance, direct-acting antiviral agents

## Abstract

The advent of direct-acting antiviral drugs (DAAs) was a breakthrough in the treatment of patients with chronic hepatitis C, yet high viral replication errors can lead to the development of resistance associated variants (RAVs). Thus, assessment of RAV in infected patients is necessary to monitor treatment effectiveness. The aim of our study was to investigate the presence of primary resistance mutations in the NS3 and NS5 regions of HCV in treatment-naive patients. Samples were taken from 42 patients with HCV who had not previously received DAA treatment. In the present study, we used the method for determining drug resistance mutations based on direct sequencing of the NS3, NS5A, and NS5B genes developed by the Saint Petersburg Pasteur Institute. Primary mutations associated with resistance were detected in 5 patients (12%). According to the Geno2pheno [hcv] 0.92 database, nucleotide substitutions were identified in various viral genes conferring resistance or decreased sensitivity to the respective inhibitors. This study has shown different mutations in the analyzed genes in patients with HCV who had not previously received DAA treatment. These mutations may increase the likelihood of treatment failure in the future.

## 1. Introduction

Hepatitis C virus (HCV) continues to be one of the main problems of world and national health care. According to the WHO, more than 170 million people in the word are affected by this virus. More than 130 thousand people die annually from chronic infection [1]. The most common complications of HCV infection are hepatocellular carcinoma and liver cirrhosis. These diseases can lead to profound disability of patients and their premature death [2].

The viral genome is translated into a polypeptide, which is sequentially processed into ten mature proteins. The structural proteins core and the envelope proteins (E1, E2) lie at the N-terminus of the polyprotein. The non-structural proteins NS3, NS4A, NS4B, NS5A and NS5B are located at the C-terminus. Between the structural and the non-structural proteins are two non-structural proteins (p7, NS2), which are dispensable for replication, but essential for assembly [3]. Core protein associates with viral RNA to form the nucleocapsid. E1 and E2 envelope glycoproteins form a heterodimer, which is most likely the functional unit of the viral envelope. The p7 polypeptide is a small hydrophobic protein, which forms an ion channel, and it is involved in viral assembly and secretion. NS2 is a multifunctional protein essential for both assembly and replication by its function as an autocatalytic cysteine protease. The N-terminal domain of NS3 is the second viral protease that processes the viral polypeptide towards the C-terminus, whereas the C-terminal domain of NS3 has a helicase function. NS4A is a small hydrophobic protein that serves as a cofactor for NS3 serine protease. NS4B protein induces the rearrangement of intracellular membranes assuring the framework for viral replication. NS5A is a multifunctional protein involved in replication and assembly. NS5B is the viral RNA-dependent RNA polymerase that forms a replication complex together with NS3, NS4A, NS4B and NS5A [4].

HCV is characterized by the highest genetic variability of the genome among all causative agents of viral hepatitis. At the moment, all HCV isolates are grouped into 7 genotypes [5]. Some subtypes are evenly distributed in the world, and the circulation of others is typical for certain regions: genotypes 1a, 1b, 3a are most common in Russia [6]. The clinical course of infection depends on several factors, including the age at the time of infection, gender, ethnicity, host genetic factors, immune status. Viral genotype, subtype and genomic variability of the virus are also significant factors [7,8].

HCV genotype and subtype determination are crucial for better understanding epidemiological and virological features of illness, including agent characteristics. Further, this provides additional information for decision making regarding antiviral therapy strategies. Viral genotypes and subtypes differ significantly concerning natural course of infection, pathogenesis, modes of transmission, disease progression, treatment regimens, responses to antiviral therapy, and clinical outcome [9].

Therapy with pegylated interferon plus ribavirin (peg-IFN-ribavirin) had been the standard of treatment for patients with chronic hepatitis C for many years. This therapy regimen had some drawbacks, such as suboptimal response to therapy in some subgroups of patients (genotype 1 and 4, severe liver fibrosis) and side effects, often resulting in poor tolerance and contraindications to treatment [10].

In 2011, a group of drugs with a completely different action appeared, replacing therapy with interferon. The targets of direct-acting antiviral drugs (DAAs) are non-structural proteins of the virus: NS3; NS5A; and NS5B. The effect of such drugs on these targets is disruption and eventual termination of viral replication [11]. 

Registered DAA regimens permit: increased therapeutic efficacy (sustained virologic response SVR > 90%), reduced frequency of side effects; and significantly simplified or shortened duration of drug administration [12]. However, the occurrence of drug resistance mutations may affect the effectiveness of DAA therapy. Due to high replication rates, the low fidelity of HCV RNA polymerase, and selective pressures by the immune system and drug treatment, there are numerous viral variants, termed quasi-species [13]. Some of these variants may have amino acid substitutions that, by changing the conformation of the drug binding site, cause resistance to therapy [14,15].

These nucleotide substitutions appear during DAA therapy and can be the cause of treatment failure (especially with first-generation drugs) [16,17]. Moreover, drug resistance mutations can also be found in treatment-naive patients [18,19,20]. Primary nucleotide substitutions associated with drug resistance occur with different frequencies and depend on the HCV genotype/subtype [21]. 

The detection of resistance associated variants (RAVs) in untreated patients has been reported internationally. Therefore, assessment of RAV in infected patients is important for monitoring treatment effectiveness and HCV epidemiology in Russia. Such studies of natural resistance mutations in untreated HCV patients may be of great importance [22,23,24,25]. The aim of this study was to investigate the prevalence of primary drug resistance mutations among patients with chronic hepatitis C (CHC) who had not previously received DAA treatment.

## 2. Materials and Methods

The study was approved by the Ethics Committee of the Saint Petersburg Pasteur Institute. The work used blood plasma of 42 patients living in the Northwestern Federal District with CHC and without a history of DAA treatment. Patient inclusion criteria for the study were: men and women; age over 18 years old; and infection occurring in the previous 12 months or less. Patient exclusion criteria for the study were: children; pregnant women; those younger than 18 years old; or infection occurring more than 12 months earlier. 

In the first stage of analysis, viral load and genotype were determined using the AmpliSense HCV-monitor-FL and AmpliSense HCV-genotype-FL reagent kits, respectively, according to manufacturer instructions. Further, RNA was obtained from blood plasma using a set of RIBO-prep reagents for the isolation of RNA/DNA from clinical material. The next step was the staging of reverse transcription. A reagent set for obtaining cDNA from RNA template, ‘REVERTA-L’ (Federal Budget Institution of Science ‘Central Research Institute of Epidemiology’, Federal Service for Surveillance on Consumer Rights Protection and Human Wellbeing, Moscow), was used.

Specific primers for each genotype were used to obtain sequences of three regions of the virus, mutations in which are associated with resistance (NS3, NS5A, NS5B). Amplification products were purified and evaluated for fragment length and concentration. Determination of nucleotide sequences was carried out using an ABI Prism 3500 genetic analyzer (Applied Biosystems, Bedford, MA, USA) according to manufacturer instructions. Primary analysis of the obtained consensus nucleotide sequences was carried out using the NCBI Blast program in comparison with the nucleotide sequences present in the international GenBank database. The resulting sequences were deposited in the international database GenBank, where they were assigned their corresponding numbers (Appendix A).

Fragments of regions NS3, NS5A, and NS5B were assessed for the presence of nucleotide substitutions leading to drug resistance mutations using the Geno2pheno [resistance] 3.4 program [26].

## 3. Results

Twenty-three patients were male, and nineteen were female. Ages ranged from 38 to 84 years old (mean age 57 years old). The results of determining viral load in patients ranged from undetectable to 1.8 × 10^8^ IU/mL, of which 33% had a viral load less than 300 IU/mL, which did not allow further analysis. Regarding HCV genotype (GT), 19 patients (68%) were infected with HCV GT1 (2 GT1a and 17 GT1b), and 9 patients (32%) were infected with GT3a (Table 1)

**Figure 1:**
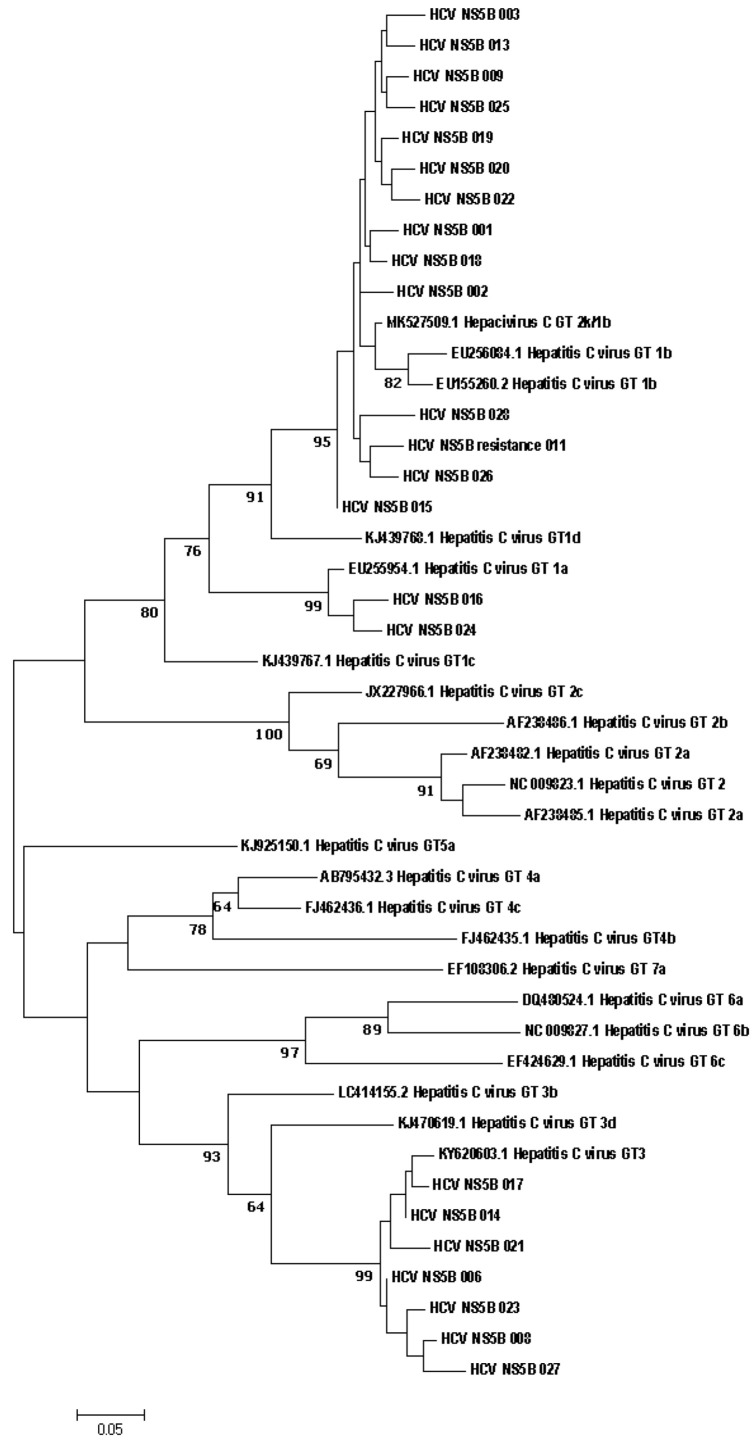
Phylogenetic analysis of the nucleotide sequences of the HCV NS5B gene fragment isolated from HCV patients from the Russian Federation in comparison with reference sequences present in GenBank. Reference sequences are designated by GenBank codes indicating the genotype. Samples studied in this work are designated by numbers. Samples from patients with resistance mutation labelled with the word ‘resistance’. Bootstrap values ≥ 60%.

The nucleotide sequences of all three regions (NS3, NS5A, NS5B) were determined in 17 samples. Due to low viral load (less than 2 × 10^3^ IU/mL), two regions were identified in 4 samples, and one region was identified in 7 samples. Phylogenetic connections between study samples obtained from patients with hepatitis C from the Russian Federation and reference sequences from the GenBank international database are shown in Figure 1. 

**Figure 2:**
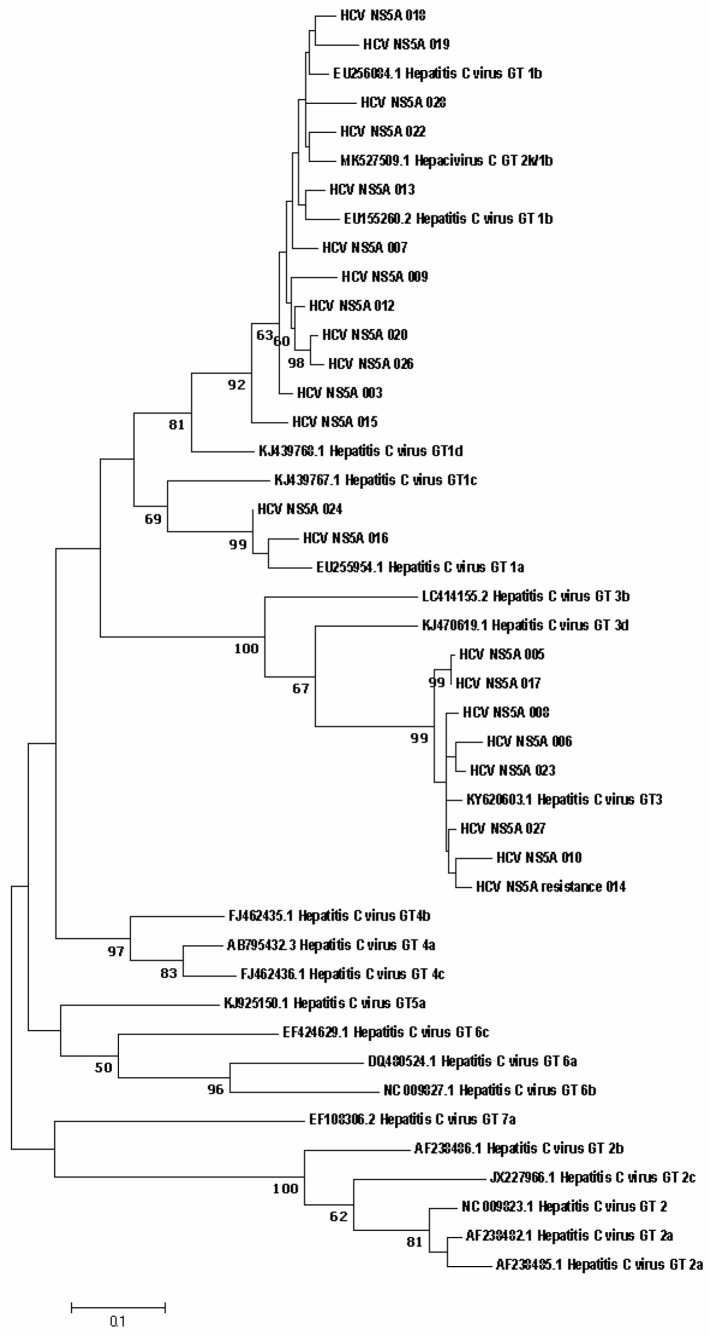
Phylogenetic analysis of the nucleotide sequences of the HCV NS5A gene fragment isolated from HCV patients from the Russian Federation in comparison with the reference sequences present in GenBank. The samples studied in this work are designated by numbers. Samples from patients with resistance mutation labelled with the word ‘resistance’. Bootstrap values ≥ 60%.

**Figure 3:**
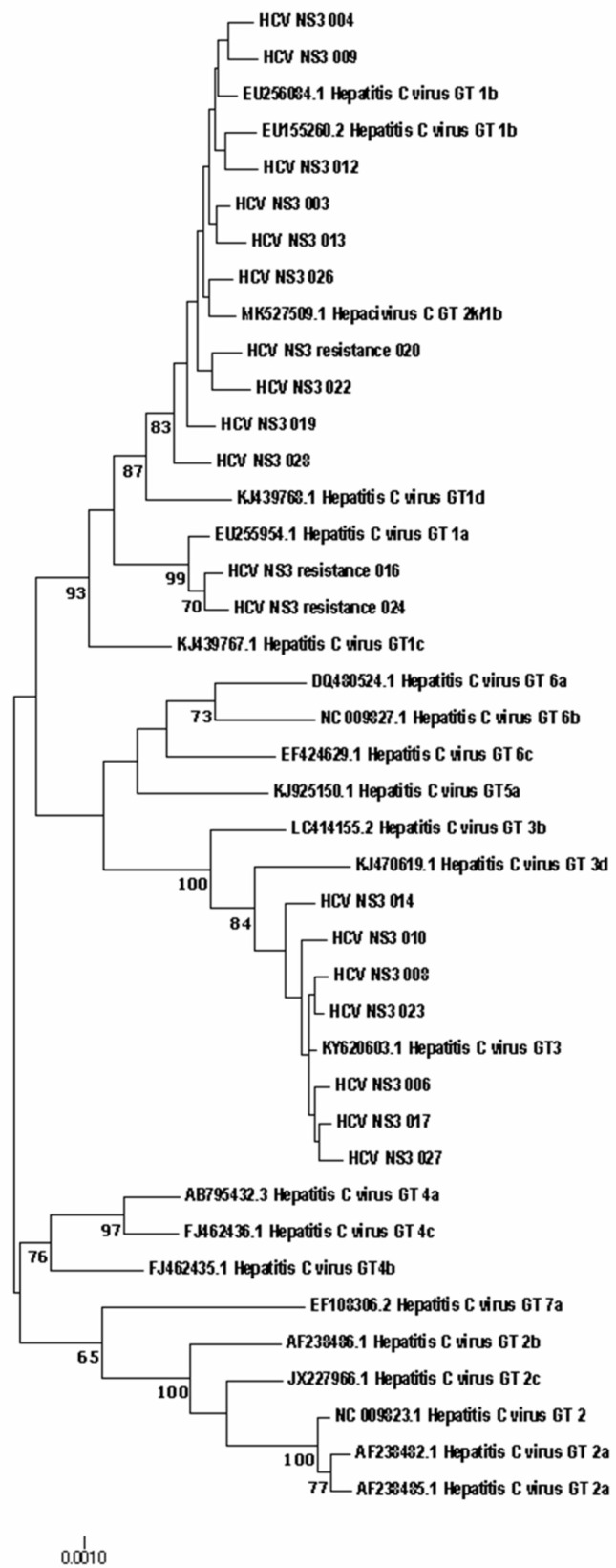
Phylogenetic analysis of the nucleotide sequences of the HCV NS3 gene fragment isolated from HCV patients from the Russian Federation in comparison with the reference sequences present in GenBank. The samples studied in this work are designated by numbers. Samples from patients with resistance mutation labelled with the word ‘resistance’. Bootstrap values ≥ 60%.

Direct sequencing of HCV region NS5B has established itself as a reliable way to identify different HCV genotypes and is considered the method of choice for characterizing viral isolates worldwide. NS5B sequence analysis for genotyping provides accurate identification of the genotype and epidemiological picture of circulating viral strains [27]. The results of genotype determination by direct sequencing do not contradict the results obtained using the ‘AmpliSense HCV-genotype-FL’ reagent kit. The phylogenetic connections between the nucleotide sequences of the NS5A and NS3 genes, obtained from HCV patients and references from the international GenBank database, are shown in Figure 2 and Figure 3, respectively.

Also, phylogenetic connections between the nucleotide sequences of the NS5a and NS3 genes obtained from HCV patients and references from the international GenBank database are shown in Figure 2 and Figure 3 respectively.

Mutations associated with HCV resistance to DAAs were found in 5 patients: 1 mutation in region NS5A; 1 mutation in region NS5B; and 3 mutations in region NS3 (Table 2).

These nucleotide substitutions have a confirmed association with DAA resistance and, if the wrong treatment strategy is chosen, can lead to therapeutic failure.

In the analyzed regions of the NS3, NS5A, and NS5B genes, a high frequency of natural polymorphism was found. Patients with HCV GT 1a in all cases encountered the following substitutions: in NS5A—K107T, S131T, E181D; in NS5B—R300K, Q309R; and in NS3—S91A, L153I.

The most common substitutions among patients with HCV GT 1b and 3a are presented in Table 3.

Among the studied isolates, several unusual mutations were recorded, including in positions characteristic of drug resistance mutations (score position): NS5A (P58S/T—14%, A62S/F—25%); NS5B (C316T/N—29%, L159P—7%, S282R—7%); and NS3 (R117Q/C—14%, V170I—21%, S122N—7%, Y56F—7%). Unfortunately, there is no information about most of them in available sources, which indicates the need to further monitor and evaluate the significance of these substitutions.

## 4. Discussion

In one patient, the Q309R substitution was found in the NS5B region associated with ribavirin resistance [28]. In some regions, both in Russia and around the world, direct antiviral therapy is not available due to high costs. For this reason, treatment regimens with interferon + ribavirin exist and are currently being used. Thus, identifying mutations associated with both direct-acting drug resistance and ribavirin resistance is important.

The increasing number of cases of recombinant HCV variants may influence the best choice of anti-HCV therapy, since pangenotypic regimens are not available to many patients due to their high cost. For optimized HCV therapy, including patients with recombinant strains, the use of genotyping assays based on a single region of the genome appears to be inappropriate. Therefore, it is recommended to use a genotyping assay that includes the 5′UTR, Core, and NS5B regions. For identification of HCV genotype, sequencing methods are suitable for the identification of recombinant HCV strains. To identify patients infected with a recombinant HCV strain, a genotyping assay using at least two different target regions at both ends of the HCV genome should be used. Treatment of patients with recombinant HCV is difficult compared with other genotypes. In such cases, it is necessary to monitor all stages of therapy to prevent failure due to the different effects of antiviral drugs on different genotypes [5,29].

Presence of the L153I substitution is not an obvious reason for a decrease in the sensitivity, or resistance of the virus, to NS3 protease inhibitors. Nevertheless, there are works evaluating this mutation as the reason for the sensitivity of the virus to the drugs of this group, and in particular to boceprevir [30]. This substitution was found in two of the patients of this study.

At the moment, not all nucleotide substitutions that lead to resistance to certain groups of drugs are known. Data on resistance mutations are steadily growing, adding to the list of significant substitutions. Thus, it is possible that not all polymorphic variants are minor. It cannot be ruled out that they might lead to future viral resistance under the influence of therapy or other factors. 

According to some reports, a nucleotide substitution leading to E237G in the NS5B region may be the reason for failure to respond to the corresponding inhibitors [31,32]. It is also known that a substitution at position 62 in the NS5A region can confer a high level of resistance to the respective inhibitors (Daclatasvir, Ledipasvir, Elbasvir, Velpatasvir) in different genotypes, but the significance of this substitution for GT3 has not yet been studied [33].

The additive effect of multiple polymorphisms in the same region continues to be studied. Thus, the S98G polymorphism, in combination with already known significant mutations, may explain a decrease or lack of response in patients with Daclatasvir therapy [34].

High genetic polymorphism is characteristic of all RNA viruses, including HCV. However, the issue of frequently encountered polymorphic variants, which can be the cause of reduced sensitivity or complete resistance to certain drugs, has not been fully studied. The importance of analyzing natural polymorphic patterns lies in their potential role as viral drug resistance mutations. Due to the emergence of new drugs and new treatment regimens, frequent nucleotide substitutions not previously associated with resistance cannot be ruled out as drug resistance mutations. DAA treatment outcome statistics continue to be updated. It is possible that reduced response or non-response to existing drugs may be due to mutations that are not currently understood to be associated with drug resistance.

Mutations that are polymorphic variants for one HCV genotype may be drug resistance mutations for another genotype. As such, even for the appointment of pan-genotypic drugs, it is necessary to determine the viral genotype to exclude the presence of mutations that may cause decreased therapeutic effectiveness in patients with that genotype.

Furthermore, the way in which these mutations interact with already known resistance associated polymorphic variants may be complex. It is theoretically possible that mutation/polymorphism combinations may confer new resistance, enhance existing resistance, or even act to make antivirals more effective. To date, there are no complete data in the literature on those polymorphic variants that were found as a result of this study. Thus, there is a need for further study of this issue.

## 5. Conclusions

The era of direct antiviral drugs holds great hopes for success in the treatment of patients with CHC. However, there are circumstances that, to one degree or another, can lead to failure of therapy or even resistance to it. The possibility of natural resistance mutations in patients significantly reduces confidence in the success of therapy. This study has shown different mutations in specific analyzed genes (NS5A, NS5B, NS3) in patients with HCV and no history of DAA treatment. Such mutations may increase the likelihood of treatment failure in the future. In our study, 18% of patients were found to have HCV mutations that can lead to a virological breakthrough, either at the beginning of therapy or at other stages.

It is very important to conduct an epidemiological analysis of the patient with HCV before initiation of therapy, and before selection of DAAs, in order to exclude the possibility of transmitting resistant viral strains. Under the pressure of antiviral therapy, the selection of resistant, quasi-species of the viral population is possible. Failure of therapy, in this case, can occur either during treatment or after, causing a recurrence of the infection.

Analyses to identify mutations in HCV conferring drug resistance to DAAs will solve these problems. Firstly, preliminary testing before prescribing therapy will enable choosing the optimal treatment regimen. Secondly, during therapy or relapse, this analysis will identify resistance mutations that could lead to therapeutic failure. Thirdly, obtaining nucleotide sequences of non-structural proteins of the virus, and further phylogenetic analysis, will enable better understanding of the epidemiological significance of the disease.

## Figures and Tables

**Table 1:** HCV genotypes in the study group.

GT	Sample Number
1a	016; 024
1b	001; 002; 003; 004; 007; 009; 011; 012; 013;015; 018; 019; 020; 022; 025; 026; 028
3a	005; 006; 008; 010; 014; 017; 021; 023; 027

**Table 2:** Mutations associated with HCV resistance to DAAs in the study group.

No. of Sample	Region	GT	Nucleotide Substitution	Inhibitor Resistance
011	NS5B	1b	L159F	Sofosbuvir
014	NS5A	3a	A30K	Daclatasvir, Elbasvir, Ledipasvir
016	NS3	1a	Q80K	Simeprevir
020	NS3	1b	Y56F	Grazoprevir
024	NS3	1a	N174S	Boceprevir

**Table 3:** The most common natural substitutions among patients with HCV GT 1b and 3a.

Region	GT 1b (N = 14)	GT 3a (N = 12)
NS3	S7A—29% A66G—57% P86Q/S—57% K87A—64% F147S—67%	I52M—67% S102A/T—50% S166A—58%
NS5A	K6R—79% S17T—67% L34V/I—79% V174S/T—67%	S14T/M/A—83% A17S/T—75% A21T—83% A62S/F—92% H85Y—50% V153L—33% L158I—33% K166R—42% D172E—75% M176S/T/A—50% H180N—42% T183A—67%
NS5B	C213S/N—50% A218S—50% S231N—50% S300T—29%	P189S—42% K304R—33% N307G—50%

## Data Availability

Publicly available datasets were analyzed in this study. This data can be found in GenBank: [EU256084.1, EU155260.2, NC_009823.1, AF238482.1, KJ439767.1, KJ439768.1, AF238485.1, AF238486.1, JX227966.1, LC414155.2, KJ470619.1, AB795432.3, FJ462435.1, FJ462436.1, KJ925150.1, DQ480524.1, NC_009827.1, EF424629.1, EF108306.2, MK527509.1].

## Appendix A. Sequences Obtained

**Sequence**	**Identifier**
HCV_NS5B_001	OM811380
HCV_NS5B_002	OM811381
HCV_NS5B_003	OM811382
HCV_NS5B_006	OM811383
HCV_NS5B_008	OM811384
HCV_NS5B_009	OM811385
HCV_NS5B_resistance_011	OM811386
HCV_NS5B_013	OM811387
HCV_NS5B_014	OM811388
HCV_NS5B_015	OM811389
HCV_NS5B_016	OM811390
HCV_NS5B_017	OM811391
HCV_NS5B_018	OM811392
HCV_NS5B_019	OM811393
HCV_NS5B_020	OM811394
HCV_NS5B_021	OM811395
HCV_NS5B_022	OM811396
HCV_NS5B_023	OM811397
HCV_NS5B_024	OM811398
HCV_NS5B_025	OM811399
HCV_NS5B_026	OM811400
HCV_NS5B_027	OM811401
HCV_NS5B_028	OM811402
HCV_NS3_003	OM811403
HCV_NS3_004	OM811404
HCV_NS3_006	OM811405
HCV_NS3_008	OM811406
HCV_NS3_009	OM811407
HCV_NS3_010	OM811408
HCV_NS3_012	OM811409
HCV_NS3_013	OM811410
HCV_NS3_014	OM811411
HCV_NS3_resistance_016	OM811412
HCV_NS3_017	OM811413
HCV_NS3_019	OM811414
HCV_NS3_resistance_020	OM811415
HCV_NS3_022	OM811416
HCV_NS3_023	OM811417
HCV_NS3_resistance_024	OM811418
HCV_NS3_026	OM811419
HCV_NS3_027	OM811420
HCV_NS3_028	OM811421
HCV_NS5A_003	OM811422
HCV_NS5A_005	OM811423
HCV_NS5A_006	OM811424
HCV_NS5A_007	OM811425
HCV_NS5A_008	OM811426
HCV_NS5A_009	OM811427
HCV_NS5A_010	OM811428
HCV_NS5A_012	OM811429
HCV_NS5A_013	OM811430
HCV_NS5A_resistance_014	OM811431
HCV_NS5A_015	OM811432
HCV_NS5A_016	OM811433
HCV_NS5A_017	OM811434
HCV_NS5A_018	OM913205
HCV_NS5A_019	OM811436
HCV_NS5A_020	OM811437
HCV_NS5A_022	OM811438
HCV_NS5A_023	OM811439
HCV_NS5A_024	OM811440
HCV_NS5A_026	OM811441
HCV_NS5A_027	OM811442
HCV_NS5A_028	OM811443
